# Unveiling the Influence and Mechanisms of Enhancing Ferrite-Phase Composition on the Properties of Calcium Sulfoaluminate Cement

**DOI:** 10.3390/ma18112457

**Published:** 2025-05-23

**Authors:** Songsong Lian, Yu Shao, Chenyu Wang, Yutian Bi, Jiaxing Ma, Kangzhan Han, Anzhe Zhu, Guogang Ying

**Affiliations:** 1 School of Civil Engineering, NingboTech University, Ningbo 315100, China; 2 School of Civil Engineering and Architecture, Zhejiang Sci-Tech University, Hangzhou 310018, China; 3 College of Architectural and Engineering, Anqing Vocational & Technical College, Anqing 246003, China; 4 Ningbo Langda Technology Co., Ltd., Ningbo 315100, China

**Keywords:** CSA, ferrite phase, C_4_A_3_Š, C_4_AF, AFt, influence mechanisms

## Abstract

Calcium sulfoaluminate (CSA) cement has emerged as a low-carbon alternative to ordinary Portland cement (OPC), offering reduced CO_2_ emissions and rapid strength development. However, the role of the ferrite phase in CSA systems remains underexplored. This study investigates the influence of ferrite-phase composition on CSA cement properties through targeted clinker design, hydration analysis, and macro–micro performance testing. Nine clinker formulations were synthesized by systematically increasing the ferrite content (10–30%) while adjusting belite (C_2_S) proportions, using limestone, bauxite, and supplementary Fe_2_O_3_/SiO_2_. Results reveal that the ferrite phase enhances the formation and stabilization of ye’elimite (C_4_A_3_Š) during clinkering and reduces low-activity transitional phase products. Increasing the iron-phase content appropriately improves early strength by promoting ettringite (AFt) formation and refines pore structures to enhance later strength development. The maximum strength improvement is achieved when the target ferrite-phase content is set to 15%, showing a 25.1% increase in 1 d strength and an 11.5% increase in 28 d strength. While ferrite phases and C_2_S ensure long-term strength gains, excessive ferrite content reduces C_4_A_3_Š availability, limiting early AFt formation and compromising initial strength. These findings highlight the dual role of the ferrite phase in optimizing CSA cement performance and sustainability, providing a foundation for designing ferrite-rich, low-carbon binders.

## 1. Introduction

The global construction industry is currently facing unprecedented challenges driven by the urgent need for sustainable development and carbon emission reduction. Ordinary Portland Cement (OPC), the most widely used binder material in modern construction, is responsible for a significant portion of anthropogenic CO_2_ emissions, estimated to be around 5–8% of the global total [1,2]. Consequently, the search for alternative, low-carbon cementitious materials has become a paramount research focus worldwide [3,4]. Among the promising alternatives to OPC, Calcium Sulfoaluminate (CSA) cement has garnered significant attention due to its distinct environmental and performance advantages [5,6]. The production of CSA clinker requires lower calcination temperatures (typically 1250–1350 °C) compared to OPC (1450 °C) and utilizes raw materials with a lower calcium carbonate content, resulting in a potential reduction of CO_2_ emissions by up to 30–40% per ton of cement produced [7,8]. Furthermore, CSA cement exhibits unique properties highly desirable for various applications, including rapid setting and hardening, high early strength development, low drying shrinkage or even micro-expansion, excellent durability particularly in sulfate-rich environments, and good performance at low temperatures [9,10,11,12].

The properties of CSA cement are primarily governed by its main mineral phases, which typically include ye’elimite (C_4_A_3_Š) and belite (C_2_S) [13,14]. C_4_A_3_Š is the principal hydraulic phase responsible for the rapid setting and early strength gain through its reaction with calcium sulfates and water to form ettringite (AFt) and aluminum hydroxide (AH_3_) [15,16]. C_2_S contributes to the later-age strength development, similar to its role in OPC [17]. Besides these major phases, CSA clinkers invariably contain ferrite phases, commonly referred to as calcium aluminoferrite (C_x_A_y_F_z_) [18]. In most conventional CSA clinkers produced using bauxite and limestone, this phase exists as a solid solution, predominantly brownmillerite (C_4_AF) [19]. The ferrite phase primarily acts as a fluxing agent during the clinkering process, facilitating the formation of the liquid phase at lower temperatures, which promotes the combination of raw materials and the formation of C_4_A_3_Š [20]. C_4_AF can also form iron gels (Fe(OH)_3_) and iron-containing hydrates (Calcium Alumino-Ferrite hydrate, C-A-F-H), which could refine pore structures, thereby improving freeze-thaw resistance and chloride impermeability [21,22]. Studies have shown that due to the presence of ferrite phase minerals, CSA cement has better resistance to chloride ion corrosion than OPC [23].

Moreover, iron-rich industrial byproducts can replace costly raw materials, aligning with green construction demands under the “dual-carbon” strategy [24,25]. Zhang and Shi et al. prepared CSA clinker using solid waste red mud as the raw material, and explored the hydration and properties of the resulting cement clinker. Results showed that compressive strength could reach 40.0 MPa and 57.9 MPa at 1 d and 28 d [26,27]. Iacobescu et al. investigated the use of electric arc furnace steel slag as a raw material in the production of calcium ferroaluminate belite cement clinker (firing at 1320 °C) to save natural resources (i.e., limestone and clays) [28].

However, the role and potential optimization of the ferrite phase in CSA cement systems have received relatively less attention compared to the extensive research focused on ye’elimite hydration and the influence of calcium sulfates. The composition of the ferrite phase can vary significantly depending on the selection of raw materials [29,30]. This variability can potentially lead to inconsistencies in cement performance [31,32]. Wu et al. found that Fe-bearing minerals play a critical role in the preparation and performance of ferrite-rich sulfoaluminate cement [33]. Recently, there is a growing recognition that simply considering the ferrite phase as an inert filler or solely a fluxing agent might overlook its potential impact on the overall performance and durability of CSA-based materials [34]. Therefore, a critical question arises: Can the performance of CSA cement be further enhanced by strategically modifying the composition and reactivity of its ferrite phase?

This research aims to systematically investigate the effect of improving the ferrite phase mineral composition on the properties of CSA cement and to elucidate the underlying mechanisms governing these effects. This study seeks to provide a fundamental understanding of the role and optimization potential of the ferrite phase in modern CSA cement technology. The findings are expected to offer valuable insights for designing advanced CSA cements with tailored properties.

## 2. Materials and Methods

### 2.1. Materials

The primary raw materials used in this study for synthesizing cement clinker were bulk industrial commodities, including limestone, bauxite, and gypsum. Their chemical compositions, determined by X-ray fluorescence (XRF) using a Malvern Panalytical Zetium instrument (Malvern, UK) on powder samples, are summarized in Table 1 and Table 2. To achieve precise adjustment of the chemical composition, the analytical-grade reagents iron oxide (Fe_2_O_3_) and silicon dioxide (SiO_2_) were employed as fine-tuning additives, ensuring stoichiometric control over the ferrite and silicate phases during clinker formation.

### 2.2. Clinker Design Methodology

CSA cement primarily comprises key mineral phases such as C_4_A_3_Š, C_2_S, and ferrite phase. In this study, the clinker composition was modified by enhancing the proportions of ferrite phase and C_2_S, based on conventional CSA cement formulations. The clinker design methodology is outlined below.

(a) Target Composition Design

Table 3 summarizes the predefined target mineralogical compositions for the cement clinker, including C_4_A_3_Š, C_2_S, ferrite phase and anhydrite (CaSO_4_, CŠ). These compositions were specifically designed to systematically investigate the influence of key phases, primarily focusing on varying the ferrite phase content. Recognizing C_4_AF as a representative ferrite-phase mineral, this study targeted a range of C_4_AF contents, from 10% up to 30%, to demonstrate how progressively higher ferrite contents influence the cement’s performance and properties. Furthermore, varying target C_2_S contents were also included in the experimental design to explore the coupled influence of ferrite and C_2_S variations on cement properties.

(b) Stoichiometric Batching Calculation

The required chemical composition of the raw mix was determined from the target clinker design values using the following governing equations (Equation (1a–e)) [35,36]. These equations are derived from the principle of mass conservation and define the total required mass of each major oxide (like CaO, SiO_2_, Al_2_O_3_, Fe_2_O_3_, SO_3_) in the raw meal as the sum of its mass contributions from the targeted clinker mineral phases. In all these equations, ω represents the mass of the corresponding substance.

ω(CaO) = 0.6512ω(C_2_S) + 0.3672ω(C_4_A_3_Š) + 0.4616ω(C_4_AF) (1a)

ω(SiO_2_) = 0.3488ω(C_2_S) (1b)

ω(Al_2_O_3_) = 0.5016ω(C_4_A_3_Š) + 0.2098ω(C_4_AF) (1c)

ω(Fe_2_O_3_) = 0.3300ω(C_4_AF) (1d)

ω(SO_3_) = 0.1311ω(C_4_A_3_Š) + 0.5882ω(CŠ) (1e)

(c) Raw Meal Proportioning

The raw material ratios (Table 4) were determined by correlating the chemical compositions of raw materials (Table 1) with the reaction-derived chemical requirements.

(d) Theoretical Clinker Yield and Basicity Coefficient Validation

According to the raw material ratio, we calculated the theoretical amount of cement clinker produced and the alkalinity coefficient (Equation (2)) [36], ensuring that the theoretical amount of cement clinker produced meets expectations and that the alkalinity coefficient was maintained at ≤1, guaranteeing optimal CaO content to fulfill the stoichiometric demands of target minerals while suppressing free lime (f-CaO) formation.

(2) $$Alkalinity\;coefficient=\frac{\omega \left(CaO\right)-0.7\omega \left({TiO}_{2}\right)}{0.73\left[\omega \left({Al}_{2}O_{3}\right)-0.64\omega \left({Fe}_{2}O_{3}\right)\right]+1.40\omega \left({Fe}_{2}O_{3}\right)+1.87\omega \left({SiO}_{2}\right)}$$

### 2.3. CSA Cement Production

**Figure 1:**
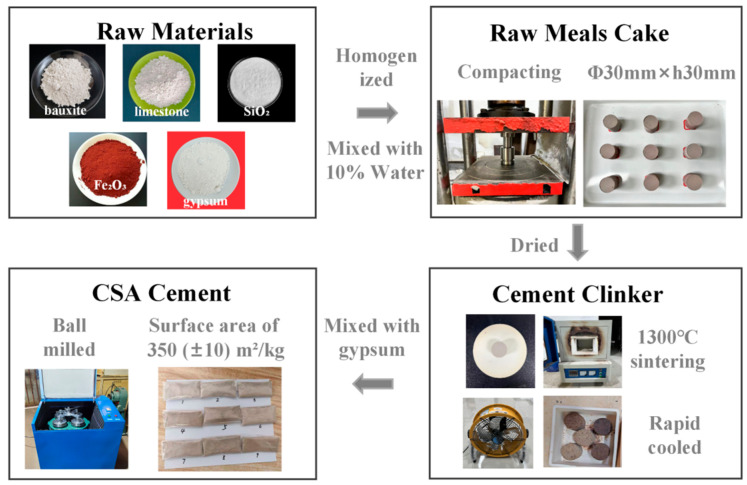
Cement production process.

The cement production process is shown in Figure 1. Raw materials were mixed and homogenized according to a predetermined ratio. The raw meal was then combined with 10 wt% water and compacted into cylindrical cakes (φ30 mm × h30 mm) under a pressure of 25 MPa. The cakes were dried at 105 °C to eliminate retained water, followed by sintering at 1300 °C in a resistance furnace for 40 min. AFter that, the sintered material was rapidly cooled using an electric fan to prevent mineral phase decomposition, obtaining CSA cement clinker. The cement clinker was subsequently mixed with 10% gypsum and ball-milled until the powder achieved a specific surface area of 350 (±10) m^2^/kg, yielding CSA cement.

### 2.4. Cement Hydration Heat Testing

The hydration heat of cement was determined using isothermal calorimetry, following the procedures outlined in ASTM C1702 [37]. Cement samples were mixed with deionized water at a water–cement ratio of 0.45 to form a homogeneous paste. This paste was then transferred into a sealed sample cell and placed within a TAM Air isothermal calorimeter maintained at a constant temperature of 20 ± 0.1 °C. Heat flow and cumulative heat release were continuously monitored for 24 h.

### 2.5. Hardened Cement Performance Testing

Cement paste specimens were prepared at a water–cement ratio of 0.45 using standardized steel cube molds (20 mm × 20 mm × 20 mm). Cement was added to the water, followed by rapid mixing for 3 min before the paste was cast into molds. Demolding was performed 24 h after casting, followed by curing in a controlled environment chamber (relative humidity ≥ 95%, temperature 20 ± 2 °C) until designated testing ages.

(a) Compressive Strength

Specimens cured for 1, 3, 7, and 28 days were subjected to compressive strength testing using a servo-controlled hydraulic testing machine (UTM-5000 series). Cubic specimens were axially loaded at a constant displacement rate of 0.6 MPa/s until failure. Triplicate samples were tested at each curing stage, with results reported as mean values ± standard deviation.

(b) X-Ray Diffraction (XRD)

Samples cured for 1 day were dried in a constant-temperature oven at 60 °C for 72 h, then pulverized and sieved through a 200 mesh (75 μm) screen. Mineralogical phase analysis was conducted using a Bruker D8 Advance X-ray diffractometer (Billerica, MA, USA) equipped with Cu-Kα radiation (λ = 1.5406 Å). Continuous scans were performed over a 2θ range of 5–90° with a step size of 0.01° and scan rate of 2°/min.

(c) Mercury Intrusion Porosimetry (MIP)

Specimens cured for 1 day were dried in a 60 °C oven for 72 h, then broken into small pieces with approximate dimensions of 5 mm^3^. A Micromeritics AutoPore IV 9600 mercury intrusion porosimeter (Micromeritics, Norcross, GA, USA) was used to analyze the porous microstructure of the fractured samples, with a pressure range of 0.1 to 60,000 psia.

(d) Scanning Electron Microscopy (SEM)

Specimens cured for 1 day were dried in a 60 °C oven for 72 h, then fractured into thin flakes with an approximate area of 5 mm × 5 mm. These flakes were mounted on aluminum stubs and sputter-coated with a thin layer of gold. The surface morphology of the samples was observed using a high-resolution scanning electron microscope (Zeiss Sigma 300, Oberkochen, Germany).

## 3. Results and Discussion

### 3.1. Clinker Composition

**Figure 2:**
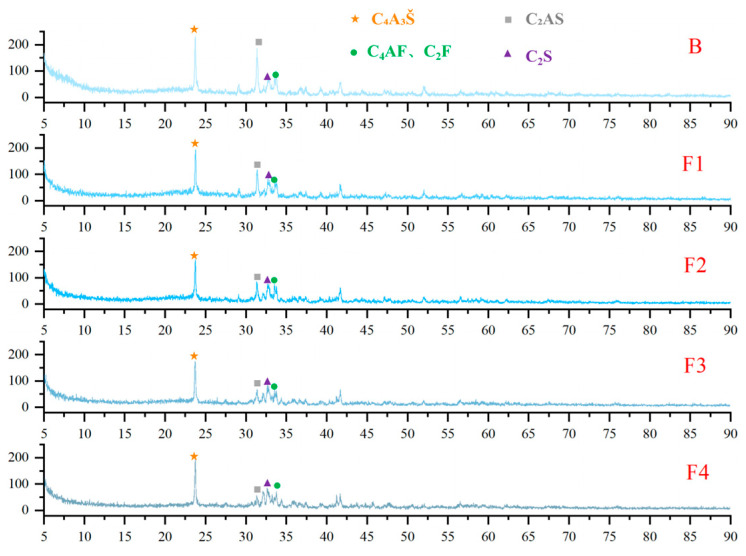
XRD patterns of cement clinkers with varying target ferrite-phase contents.

**Figure 3:**
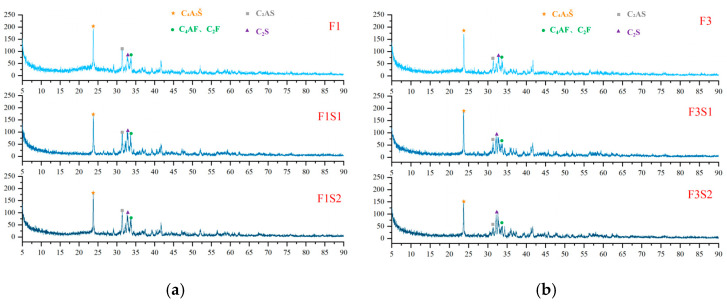
XRD patterns of cement clinkers under fixed target ferrite-phase content but varying C_2_S contents: (**a**) Target ferrite phase of 15%; (**b**) Target ferrite phase of 25%.

**Figure 4:**
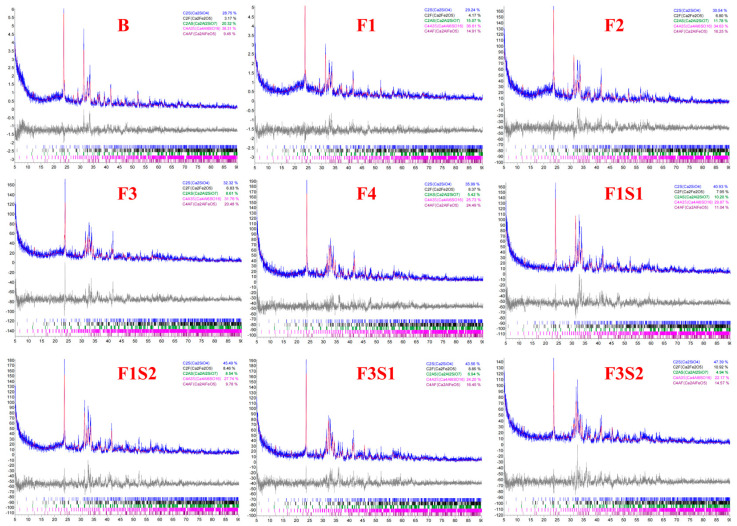
Quantitative phase analysis patterns of samples.

XRD analysis was conducted on cement clinkers prepared under different compositional targets, including varying ferrite-phase contents and varying C_2_S contents (Figure 2 and Figure 3). Across all samples, the primary mineral phases consistently identified were C_4_A_3_Š, C_2_S, and the ferrite phase, including C_4_AF and dicalcium ferrite (C_2_F, Ca_2_Fe_2_O_5_). Additionally, the metastable intermediate phase gehlenite (C_2_AS, Ca_2_Al_2_SiO_7_), a low-reactivity transitional product, was observed in certain samples. Comparative analysis of diffraction peak intensities confirmed that the experimental mineral assemblages generally aligned with the designed targets. To compare the variations in mineral phases among the groups, quantitative analysis was performed using TOPAS-Academic (Bruker, Ettlingen, Germany). The fitting index (Rwp) for all refinements was controlled to be below 10%. Analysis patterns are shown in Figure 4.

**Figure 5:**
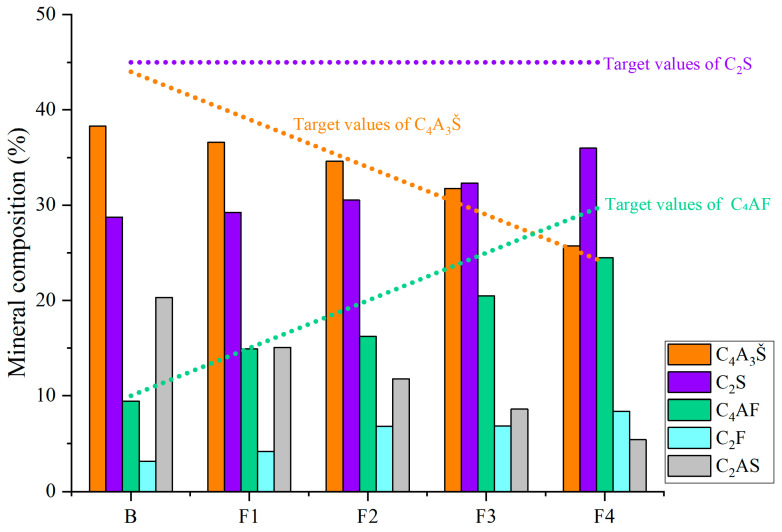
Mineral composition of cement clinkers with varying target ferrite-phase contents.

**Figure 6:**
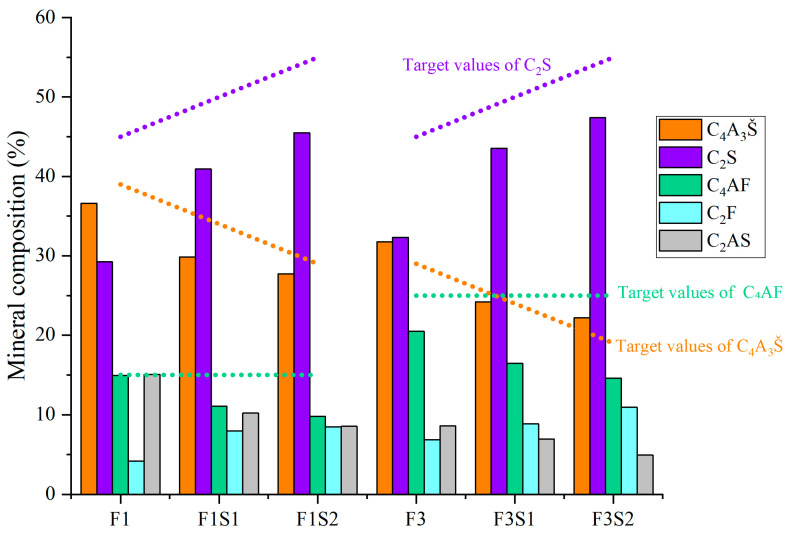
Mineral composition of cement clinkers under fixed target ferrite-phase content but varying C_2_S contents.

Based on the quantitative analysis patterns shown in Figure 4, the proportion of each mineral was statistically analyzed, and the results are presented in Figure 5 and Figure 6. Figure 5 shows the mineral composition of cement clinkers with varying target ferrite-phase contents. Specifically, it was observed that at lower target ferrite-phase contents, the C_4_A_3_Š content was consistently lower than the target values. As the target ferrite-phase content increased, the measured C_4_A_3_Š content progressively approached the target design value. This trend suggests that elevated ferrite-phase content enhances the formation and development of C_4_A_3_Š. Notably, when the target ferrite-phase content exceeded 20%, the determined C_4_A_3_Š content was slightly higher than the target value. This could potentially be attributed to the formation of a C_4_A_3−x_F_x_Š solid solution during clinker burning, involving the substitution of aluminum by iron atoms within the C_4_A_3_Š structure [38,39,40]. Simultaneously, analysis of the C_4_AF content across the different groups revealed that the experimentally determined values were consistently lower than their target values. This discrepancy may be primarily due to two factors: firstly, the formation of other iron-bearing mineral phases such as C_2_F, whose content increased with increasing target ferrite phase content; and secondly, iron entering the C_4_A_3_Š phase, as mentioned previously. Furthermore, at lower ferrite-phase contents, the C_2_S content was lower than the target value, and the content of the C_2_AS was relatively high. Conversely, as the target ferrite-phase content increased, the C_2_S content approached the target value, while the C_2_AS content progressively decreased. This observation indicates that the presence of the ferrite phase promotes the formation and stabilization of C_2_S. Mechanistically, during clinkering, the ferrite phase acts as a flux, lowering the eutectic temperature of the raw mix and facilitating the formation of the crystalline structure [41]. In summary, ferrite phases in CSA cement clinker primarily exist as C_4_AF and C_2_F. The increase in target ferrite-phase content is beneficial for the formation and stability of both C_4_A_3_Š and C_2_S. This trend underscores the critical role of ferrite-phase content in modulating competitive phase equilibria and suppressing potentially deleterious intermediate phases like C_2_AS, providing actionable insights for optimizing raw mix design in industrial cement production.

Figure 6 shows mineral composition of cement clinkers under fixed target ferrite-phase content but varying C_2_S contents. With the target ferrite-phase content fixed, an increase in the target C_2_S content leads to a significant decrease in the actual amount of C_4_AF formed in the synthesized clinker, gradually deviating from the initially set target value. Concurrently, this is accompanied by an increased formation of low-activity C_2_F. This phenomenon suggests that simply increasing the target C_2_S content can interfere with the normal formation of C_4_AF, resulting in the formation of the less reactive C_2_F instead of the more reactive C_4_AF from the raw materials. Given the hydration activity, an increase in C_2_F content negatively impacts clinker performance. Therefore, it is crucial to carefully consider the influence of C_2_S content adjustments on the ferrite-phase composition and to precisely control the target value of C_2_S during the synthesis process.

### 3.2. Hydration Characteristics

**Figure 7:**
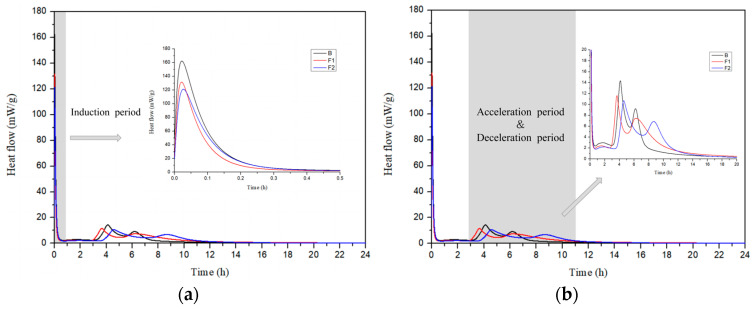
Hydration heat flow of cement with different target ferrite phases: (**a**) induction period; (**b**) acceleration/decay period.

**Figure 8:**
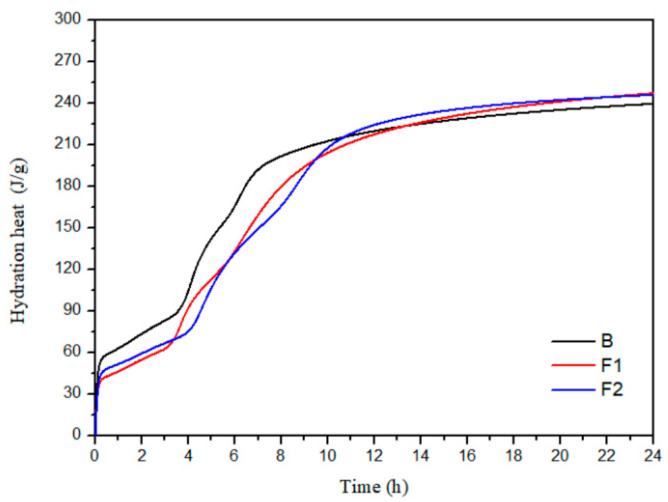
Cumulative hydration heat of cement with different target ferrite phases.

The hydration heat release process of cement samples B, F1, and F2 with different ferrite-phase contents was tested. Figure 7 and Figure 8 show the hydration heat flow curves and cumulative heat release curves of the cement with different target iron phases. From Figure 8, it can be seen that the total heat release of the three groups of samples in this study is relatively close, all around 240 J/g after 1 day.

As shown in Figure 7a, the heat flow curve of the samples has three heat release peaks. The first heat release peak is mainly due to the heat released from the dissolution of clinker particles, accompanied by the rapid hydration of a small amount of sulfoaluminate phase in the clinker with gypsum, marking the induction period of cement hydration [42,43]. Comparing the first heat release peaks of different target ferrite contents, it can be observed that as the target ferrite content increases, the first heat release peak decreases. This is because the iron solid solution and iron-alumina phase in the clinker minerals dissolve more slowly compared to the sulfoaluminate phase, resulting in a lower dissolution peak. The induction period for the three samples is similar, all ending within 1 h.

According to Figure 7b, the samples exhibit a second and a third heat release peak, corresponding to two acceleration/decay periods of the cement. The second heat release peak occurs around 4 h, primarily due to the reaction with gypsum to form AFt, accompanied by the hydration of some ferrite phase minerals [44,45]. Around 6 h, the third heat release peak appears, mainly due to the further hydration of C_4_A_3_Š and ferrite phase minerals. Comparing the heat release rates of different samples, it can be found that as the target ferrite content increases, the heat release rate decreases, and the duration of the second heat release peak increases. This may be because the increase in ferrite-phase content is accompanied by a decrease in the target design value of C_4_A_3_Š, thereby resulting in a reduction in heat flow during the first acceleration phase and an increase in the duration of the second acceleration phase.

### 3.3. Cement Strength Development

**Figure 9:**
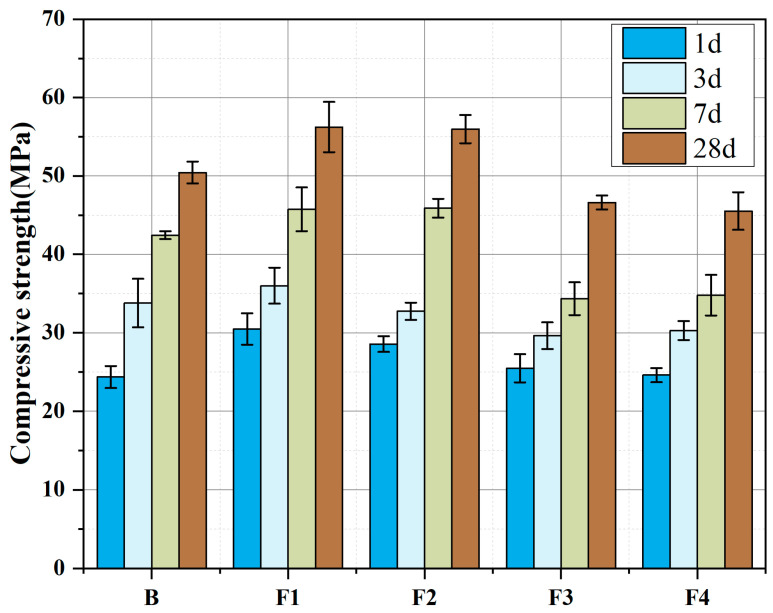
Compressive strength development of cements with varying target ferrite-phase contents.

Figure 9 illustrates the compressive strength evolution of cements with varying target ferrite-phase contents at 1 d, 3 d, 7 d, and 28 d. All five groups exhibit excellent early and long-term strength performance, characterized by high early-age strength: 1 d compressive strength exceeds 24.0 MPa (maximum 30.0 MPa). This is attributed to rapid hydration of C_4_A_3_Š or ferrite-phase minerals and gypsum, promoting swift ettringite (AFt) formation that reinforces the hardened paste. Long-term strength progressively increases, reaching >50.0 MPa at 28 d, driven by sustained hydration of ferrite phases and C_2_S.

Comparing the strength of samples with different target ferrite-phase contents, it can be observed that as the target ferrite-phase content increases, the overall strength shows a trend of first increasing and then decreasing. This is because, when the ferrite-phase content is within an appropriate range, the presence of the ferrite phase can ensure the development and stability of C_4_A_3_Š and C_2_S during clinkering, maintaining the content of active minerals. The maximum strength improvement is achieved when the target ferrite-phase content is set to 15%, showing a 25.1% increase in 1 d strength and an 11.5% increase in 28 d strength. However, when the ferrite-phase content is too high, the increase in low-activity C_2_F may negatively affect the strength development of the samples. This study suggests that the reasonable target ferrite-phase range is between 15% and 20%.

Additionally, it can be observed that the growth of compressive strength from 1 day to 3 days is relatively gentle, with a low growth rate, while the strength growth from 3 days to 28 days is significant, and the growth rate of strength at 28 days shows an increasing trend with the increase in ferrite-phase content. This indicates that the strength from 1 day to 3 days mainly depends on the content of early-strength minerals; after 3 days, the ferrite-phase minerals and C_2_S gradually participate in the reaction, and the development of strength at 28 days should primarily depend on the hydration of C_4_AF and C_2_S.

**Figure 10:**
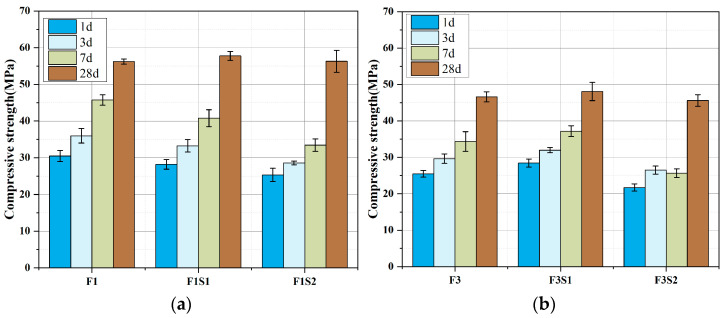
Compressive strength development of cements with fixed target ferrite-phase content but varying C_2_S contents: (**a**) target ferrite phase of 15%; (**b**) target ferrite phase of 25%.

Figure 10 shows the compressive strength of cement with varying C_2_S contents at 1 day, 3 days, 7 days, and 28 days. As can be seen from the figure, increasing C_2_S content leads to a decrease in early strength (in the first 7 days), which is due to the reduced content of C_4_A_3_Š in the cement, resulting in a lower amount of AFt formation that provides early strength. However, the later strength development rate is relatively fast, indicating that the high content of C_2_S has a significant effect on later strength.

### 3.4. Microstructural Analysis

Hardened cement specimens cured for 1 day were subjected to microstructural characterization, including hydration product composition, microporous structure, and microscopic morphology.

#### 3.4.1. Hydration Product Composition

Based on the composition of high-ferrite CSA, C_4_A_3_Š and C_4_AF can react with gypsum (CaSO_4_·2H_2_O, CŠH_2_) respectively to form ettringite (AFt), aluminum gel (Al(OH)_3_, AH_3_), and iron gel (Fe(OH)_3_, FH_3_). The hydration reactions are proposed as follows (Equations (3a,b)) [21,45,46,47]:

(3a) $$C_{4}A_{3}Š+CŠH_{2}+H\to Aft+AH_{3}(gel)$$

(3b) $$C_{4}AF+CŠH_{2}+H\to Aft+FH_{3}(gel)$$

**Figure 11:**
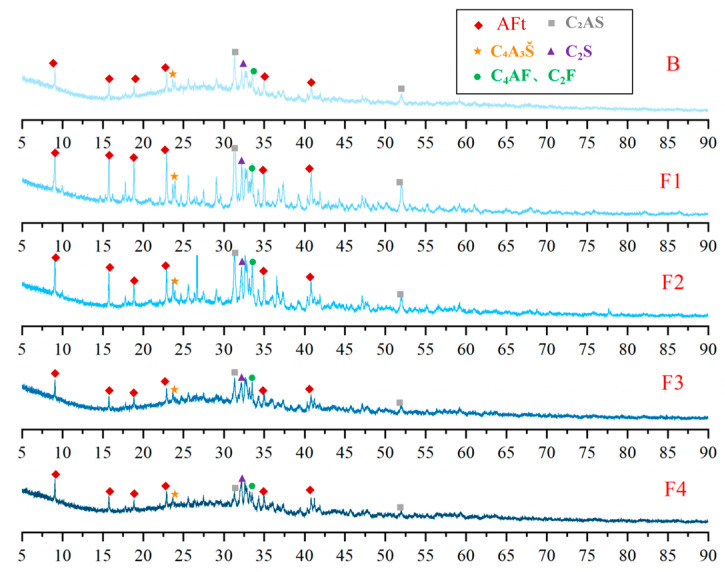
XRD patterns of hydration products at 1 day with varying target ferrite-phase contents.

**Figure 12:**
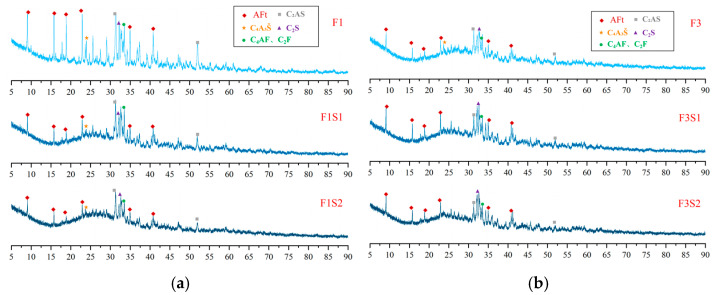
XRD patterns of hydration products at 1 day with fixed target ferrite-phase content but varying C_2_S contents: (**a**) target ferrite phase of 15%; (**b**) target ferrite phase of 25%.

XRD patterns of hydration products at 1 day (Figure 11 and Figure 12) confirm that AFt is the dominant crystalline phase across all samples, consistent with theoretical predictions. Meanwhile, a prominent amorphous halo is consistently observed in the XRD patterns of all samples, indicating the presence of a considerable quantity of non-crystalline or poorly crystalline material. Considering the well-established tendency of Al(OH)_3_ and Fe(OH)_3_ to form as amorphous gels during hydration [48], their presence likely contributes significantly to this observed diffuse scattering signal. Additionally, unhydrated clinker phases—such as C_4_A_3_Š, C_2_S, C_4_AF, C_2_F and C_2_AS—were identified, reflecting incomplete hydration at this early stage. Comparative analysis of clinker and hydration product XRD patterns reveals a significant reduction in C_4_A_3_Š content, whereas C_2_AS remains largely unchanged due to its low reactivity. Notably, the AFt content exhibits a non-linear dependence on ferrite content: it initially increases with moderate ferrite elevation (≤20%) but declines at higher levels (>20%). This trend correlates with the reduced C_4_A_3_Š content in high-ferrite clinkers, thereby limiting the sulfate–aluminate reaction essential for AFt formation.

Figure 12 presents the XRD patterns of hydration products at 1 day for samples with a fixed target ferrite-phase content but varying C_2_S contents. As shown in the figure, increasing the target C_2_S content appears to have minimal influence on the types and proportions of the hydration products at this early age. This observation is likely attributable to the relatively slow hydration rate of C_2_S, as indicated by the persistently high diffraction peaks of unreacted C_2_S visible in the patterns. Furthermore, with increasing target C_2_S content, the hydration of the C_4_A_3_Š phase becomes progressively more complete. Notably, in the F3S2 sample, the diffraction peaks of C_4_A_3_Š are almost undetectable. This trend highlights that the early strength development of these CSA-based systems is predominantly driven by the hydration products of C_4_A_3_Š, primarily AFt. Conversely, C_2_S is likely to contribute more significantly to the later-age strength of the material.

#### 3.4.2. Microporous Structure

**Figure 13:**
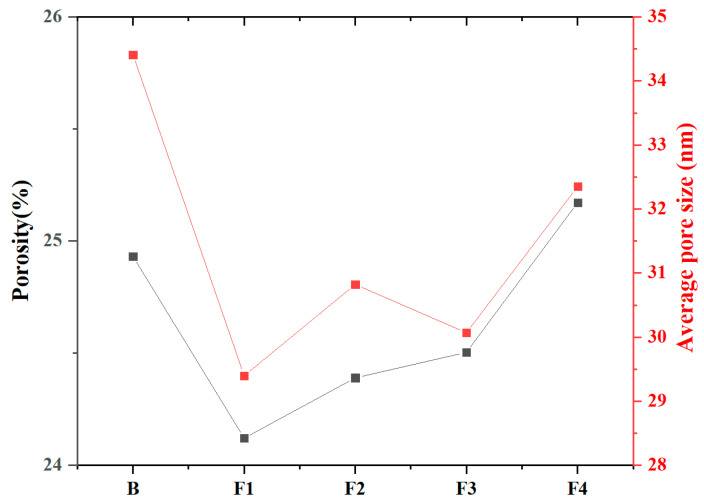
Porosity and average pore size of samples at 1 day hydration.

**Figure 14:**
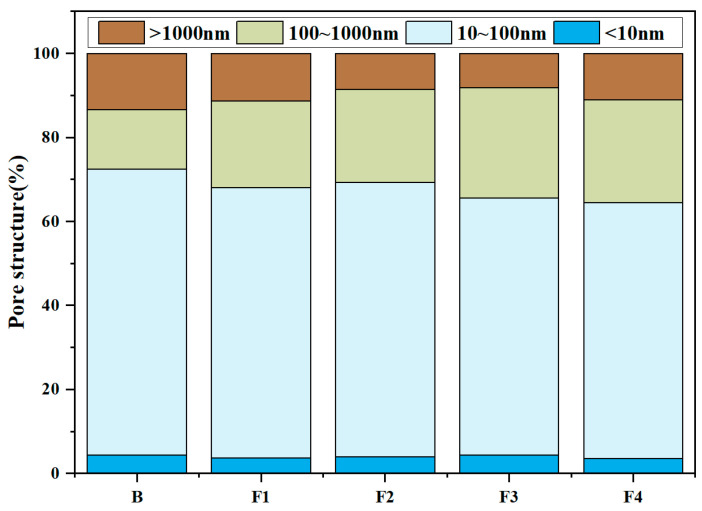
Pore structure of samples at 1 day hydration.

MIP tests were conducted to quantify the porosity, average pore diameter, and pore structure of samples at 1 day hydration (Figure 13 and Figure 14). Results reveal total porosity and average pore diameter initially decrease with increasing target ferrite content but rebound at higher levels (>15%). Notably, the sample with 15% target ferrite content (Group F1) achieves optimal pore refinement, exhibiting the lowest porosity (24.1%) and smallest average pore diameter (29.4 nm). This trend is attributed to the dual role of ferrite phases: (1) moderate target ferrite content (≤15%) enhances micro-aggregate filling effects, refining pore size distribution and reducing connectivity via dense packing of nano-scale C_4_AF particles; (2) excessive target ferrite content (>15%) impedes early hydration reactions, inducing localized porous structures due to incomplete dissolution of Fe-rich phases and interfacial debonding at boundaries. Concurrently, the proportion of pores >100 nm increases slightly with ferrite elevation, reflecting compromised microstructure homogeneity under high-ferrite conditions.

#### 3.4.3. Microstructural Morphology

**Figure 15:**
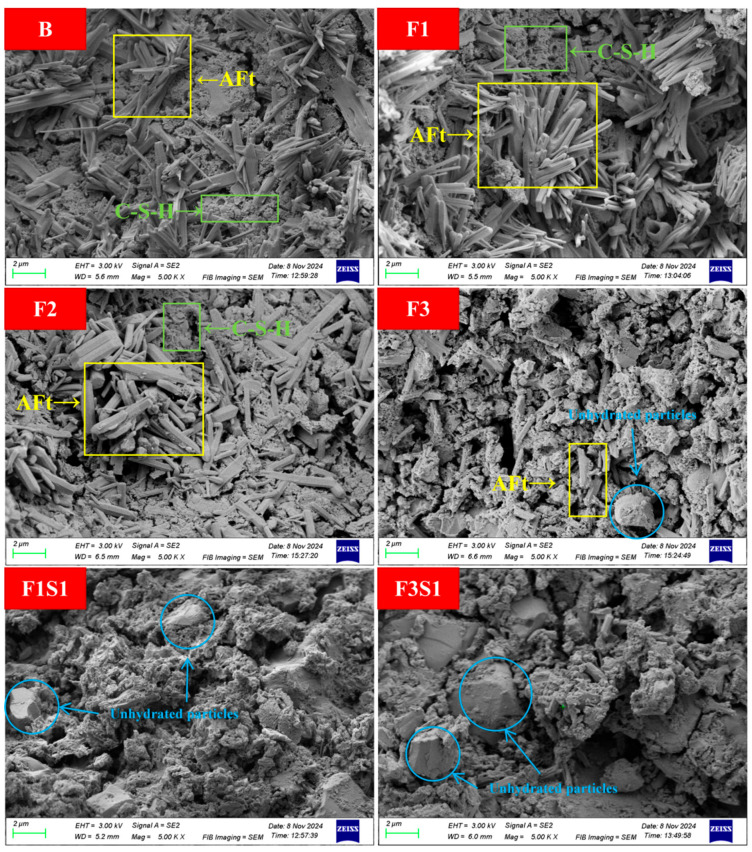
SEM images of samples after 1 day hydration.

Figure 15 illustrates the SEM microstructural characteristics of samples after 1 day hydration. In experimental groups B, F1, and F3, abundant needle-like or rod-shaped ettringite (AFt) crystals with smooth surfaces, sharp boundaries, and uniform geometric dimensions (length: 1–2 µm; diameter: 0.1–0.3 µm) are clearly observed. These AFt crystals exhibit typical radial or clustered growth patterns, forming an interlocked structure with amorphous or fibrillar calcium silicate hydrate (C-S-H) gel. Specifically, the AFt crystalline framework is embedded within the flocculated C-S-H matrix, collectively constructing a dense early-stage hydration network that contributes to superior initial mechanical strength [49,50]. Notably, the AFt crystals in groups F1 and F3 demonstrate significantly larger dimensions (up to 2.5 µm in length), higher spatial density, and more pronounced clustering compared to group B, suggesting that optimized iron content enhances AFt crystal growth and stabilization.

In contrast, AFt crystal abundance is drastically reduced and spatially dispersed, while the C-S-H gel transitions from dense agglomerates to a loose network in groups F1S1, F3S1, and F3S2. Additionally, irregular smooth-surfaced particles with sharp edges (average size: 1.5–3 µm) are distributed throughout the matrix, identified as unreacted ferrite (C_4_AF) and C_2_S particles from the clinker. This microstructural evolution indicates that excessive ferrite and C_2_S content reduces early (1 d) hydration kinetics, delays reaction progress, and retards hydration product formation—a conclusion consistent with the mechanical performance data. These findings further validate the pivotal role of C_4_AF and C_2_S in modulating early hydration behavior, highlighting the need for balanced stoichiometric control in cement formulation.

## 4. Conclusions

This study demonstrates the critical impact of enhancing ferrite-phase composition on the properties of CSA cement, with key findings as follows:

1. The ferrite phase plays a critical role in regulating the mineralogical evolution of CSA clinker. Higher ferrite-phase content helps C_4_A_3_Š and C_2_S align more closely with their corresponding target design values, demonstrating that the ferrite phase promotes their stabilization during clinkering while reducing low-activity transitional products like C_2_AS.
2. The hydration characteristics of CSA cements are modulated by ferrite-phase content as evidenced by three distinct heat release peaks. Increasing ferrite phase suppresses the initial dissolution peak and prolongs the secondary reaction stage (4–6 h) by reducing AFt formation rates as ferrite phases partially replace sulfoaluminate components.
3. Moderate ferrite-phase content (15–20%) optimizes the strength development of calcium sulfoaluminate (CSA) cement: Early-stage strength (1–3 days) improves due to the ferrite phase (C_4_AF) promoting rapid nucleation of ettringite (AFt), while later-stage strength (28 days) benefits from pore structure refinement through secondary reaction products of the ferrite phase, achieving a balance between early hydration activity and long-term microstructural optimization.
4. Excessive ferrite content (>25%) causes adverse effects in the strength development of CSA: During clinkering, an elevated Fe_2_O_3_/Al_2_O_3_ ratio reduces C_4_A_3_Š formation and limits early AFt production, while increased proportions of low-reactivity C_2_F further degrade performance.

## Figures and Tables

**Table 1:** Principal chemical compositions of raw materials (wt%).

	CaO	SiO_2_	Al_2_O_3_	Fe_2_O_3_	MgO	TiO_2_	SO_3_	Loss
Limestone	51.16	2.26	0.74	0.41	4.16	-	0.11	40.55
Bauxite	0.81	30.89	46.51	6.12	0.17	2.23	0.11	12.13
Gypsum	28.71	1.93	0.90	0.29	0.73	0.03	43.98	22.30

**Table 2:** Trace element composition of raw materials (wt%).

	P_2_O_5_	Cl	Cr_2_O_3_	MnO	CuO	SrO	ZrO_2_
Limestone	0.0553	0.0499	-	0.0196	-	0.0125	-
Bauxite	0.1441	0.0202	0.0290	0.0114	0.0053	0.0202	0.0457
Gypsum	0.0202	0.0855	-	-	-	0.0171	-

**Table 3:** Target mineralogical compositions for the cement clinker (%).

Samples	C_4_A_3_Š	C_2_S	C_4_AF	CŠ
B	44	45	10	1
F1	39	45	15	1
F2	34	45	20	1
F3	29	45	25	1
F4	24	45	30	1
F1S1	34	50	15	1
F1S2	29	55	15	1
F3S1	24	50	25	1
F3S2	19	55	25	1

**Table 4:** Raw material ratios for experimental samples (%).

Samples	Limestone	Bauxite	SiO_2_	Fe_2_O_3_	Gypsum
B	57.48	33.27	0	0	9.25
F1	58.71	31.32	0.39	1.26	8.32
F2	59.86	29.32	1.02	2.44	7.36
F3	61.01	27.32	1.64	3.62	6.41
F4	62.17	25.31	2.26	4.81	5.45
F1S1	60.85	27.78	2.57	1.47	7.34
F1S2	62.97	24.26	4.74	1.67	6.36
F3S1	63.14	23.78	3.82	3.82	5.43
F3S2	65.26	20.27	5.98	4.02	4.47

## Data Availability

The original contributions presented in this study are included in the article. Further inquiries can be directed to the corresponding author.
